# Prevalence of lower limb deep venous thrombosis among adult HIV positive patients attending an outpatient clinic at Mulago Hospital

**DOI:** 10.1186/s12981-018-0191-1

**Published:** 2018-01-25

**Authors:** Sosthene Tsongo Vululi, Samuel Bugeza, Muyinda Zeridah, Henry Ddungu, Akello Betty Openy, Mubiru Frank, Rosalind Parkes-Ratanshi

**Affiliations:** 1 Makerere University College of Health Sciences (MakCHS), Kampala, Uganda; 2 Radiology Department of Mulago Hospital, Lecturer at MakCHS, Kampala, Uganda; 3 Mulago Hospital, Kampala, Uganda; 4 Uganda Cancer Institute, Kampala, Uganda; 5 MakCHS, Kampala, Uganda; 6 Stastician-IDI-Kampala, Kampala, Uganda; 7 Prevention Care Treatment, Infectious Diseases Institute (IDI), Kampala, Uganda

**Keywords:** Lower limb veins anatomy, Well’s score, Doppler ultrasound, DVT echo pattern

## Abstract

**Background:**

Deep venous thrombosis (DVT) and its major complication pulmonary embolism (PE) are collectively known as venous thromboembolism. In Uganda, the prevalence of DVT among HIV patients has not been previously published. The aim of the study was to determine the prevalence and sonographic features of lower limb deep venous thrombosis among HIV positive patients on anti-retroviral treatment (ART).

**Methods:**

This was a cross sectional study in which HIV positive patients on ART were recruited from an out-patient HIV clinic at Mulago National Referral Hospital. Patients were randomly selected and enrolled until a sample size of 384 was reached. Study participants underwent compression and Doppler ultrasound studies of both lower limb deep veins using Medison Sonoacer7 ultrasound machine.

**Resuts:**

We found a prevalence of DVT of 9.1% (35 of 384 participants) among HIV patients on ART. The prevalence of latent (asymptomatic) DVT was 2.3%. Among 35 patients with DVT, 42.8% had chronic DVT; 31.1% had acute DVT and the rest had latent DVT. Among the risk factors, the odds of occurrence of DVT among patients with prolonged immobility were 4.81 times as high as in those with no prolonged immobility (p = 0.023; OR = 4.81; 95% CI 1.25–18.62). Treatment with second line anti-retroviral therapy (ART) including protease inhibitors (PIs) was associated with higher odds of DVT occurrence compared with first line ART (p = 0.020; OR = 2.38; 95% CI 1.14–4.97). The odds of DVT occurrence in patients with a lower CD4 count (< 200 cells/µl) were 5.36 times as high as in patients with CD4 counts above 500 cells/µl (p = 0.008). About 48.6% patients with DVT had a low risk according to Well’s score.

**Conclusion:**

DVT was shown in nearly 10% of HIV patients attending an out-patient clinic in an urban setting in Uganda. Risk factors included protease inhibitors in their ART regimen, prolonged immobility, and low CD4 count (< 200 cells/µl). Clinicians should have a low threshold for performing lower limb Doppler ultrasound scan examination on infected HIV patients on ART who are symptomatic for DVT. Therefore, clinicians should consider anti-coagulant prophylaxis and lower deep venous ultrasound screening of patients who are on second line ART regimen with low CD4 cell counts and/or with prolonged immobility or hormonal contraception.

## Background

Deep venous thrombosis is one of the most prevalent medical conditions [1]. The risk of DVT in the general population of South Africa is 0.10% a year [2]. HIV infection has been recognised as a hypercoagulable condition since the late 1980s and the current and other studies indicate that the prevalence in HIV positive patients is significantly increased [3, 4] with a two to tenfold increased risk in HIV infected patients compared to the general population. A large number of worldwide studies reported the frequency of DVT in HIV-infected patients ranging from 0.19 to 8% [5]. There is some evidence to suggest that anti-retroviral therapy (ART) may increase the risk of DVT [6]. There is limited work on DVT in sub-Saharan Africa, especially Uganda. A study done by Mangeni et al. in 2003 at Mulago Hospital showed that out of 86 patients clinically suspected to have lower limb DVT, 38 (44.2%) were found to have DVT after sonography [7]. Doppler ultrasound of lower limb deep veins presents an echo-pattern of the thrombus usually found in DVT. The thrombus may be anechoic, hypoechoic, heterogeneous or hyper-echoic depending on the age of the clot. To our knowledge, there has been no study conducted in Eastern Africa to determine the prevalence of DVT in HIV positive patients. The aim of this study was to determine the prevalence of DVT in HIV outpatients on ART in Uganda and to appreciate the association between clinical presentation and sonographic features of DVT.

## Materials/methods

This study was conducted from May 2014 to January 2015. This was a cross sectional study in which adult HIV positive outpatients on ART were recruited from the Infectious Diseases Institute and HIV clinic at Mulago Hospital. Participants were attending for routine follow-up of their HIV. Participants unable to understand the procedure and unable to consent for ultrasound scan examination were excluded. Using systematic sampling [8], patients who met the inclusion criteria were enrolled until the sample size of 384 was reached. Informed consent was signed by each participant before ultrasound scan examination. Study participants underwent Doppler ultrasound studies of both lower limb deep veins using an ultrasound machine (Medison Sonoacer7).

The patients were categorized depending on the ultrasound findings: latent DVT when there was lack of venous compressibility or reduction of blood flow on ultrasound; acute DVT was diagnosed in the presence of hypoechoic thrombus with limited venous compressibility; chronic DVT when there was hyper echoic or heterogeneous thrombus and limitation of venous compressibility. Proximal DVT was diagnosed in the presence of a thrombus in common femoral vein up to popliteal vein whereas distal DVT was seen below the popliteal fossa. A Wells score was calculated for each patient based on the presence of clinical symptoms. At the time of the study, WHO and Ugandan guidelines recommended first line ART to be backbone of nucleoside reverse transcriptase inhibitors (lamivudine/zidovudine or lamivudine/tenofovir) with either nevirapine or efavirenz non-nucleoside reverse transcriptase inhibitors. Second line regimens for those who had failed first line was either of these backbones with a protease inhibitor; either ritonavir boosted lopinavir or ritonavir boosted atazanavir. A structured non-disguised questionnaire was used and acquired data were entered using EPI—DATA 3.02 and exported to be analysed using a statistical software program STATA 13.

The study was approved by the IDI Scientific Review Committee (SRC) and the School of Medicine Research and Ethics Committee (SOMREC).

## Results

Three hundred eighty-four patients were enrolled. 286 participants were aged between 18 and 50 years (74.5%) and the remainder were above 50 years (25.5%). The most common occupation in 38.5% of participants was business (see Table 1).

**Table 1:** Study participants by gender, age-group categories and occupation

Demographics	Number (n)	Percentage (%)
Gender		
Male	126	32.8
Female	258	67.2
Age (years)		
18–29	40	10.4
30–39	110	28.7
40–49	136	35.4
≥ 50	98	25.5
Occupation		
Business	148	38.5
Driver	18	4.7
Farmer	43	11.2
House wife	28	7.3
Work in office	30	7.8
Others	117	30.5

Out of 384 study participants, 229 (59.6%) were on first line ART regimen and 155 (40.4%) participants were on second line.

**Fig. 1:**
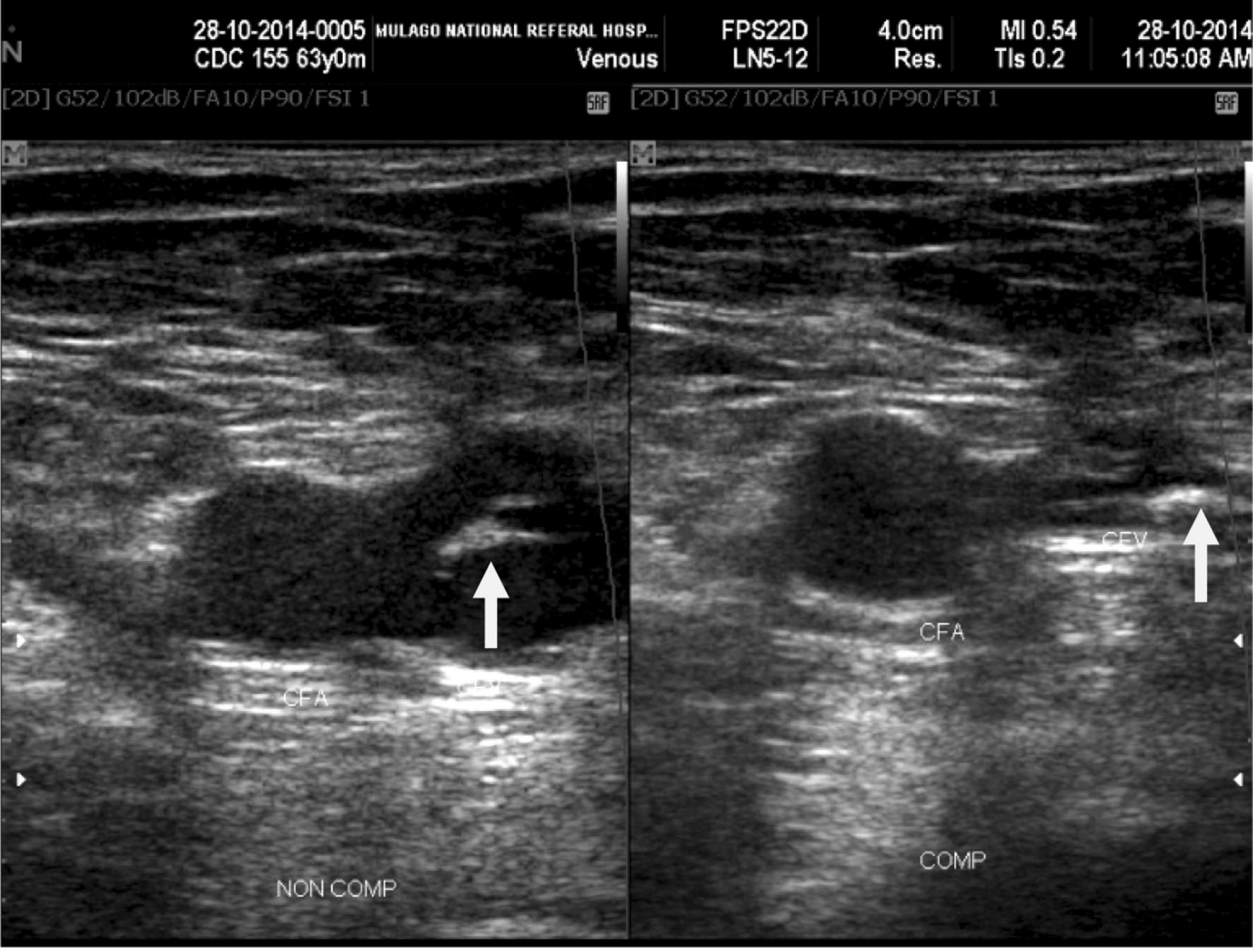
Chronic DVT. Transverse sonogram of 63 years old male HIV positive patient on HAART shows echogenic thrombus (white arrow) in the right common femoral vein (CFV) causing partial compressibility of the vein

**Fig. 2:**
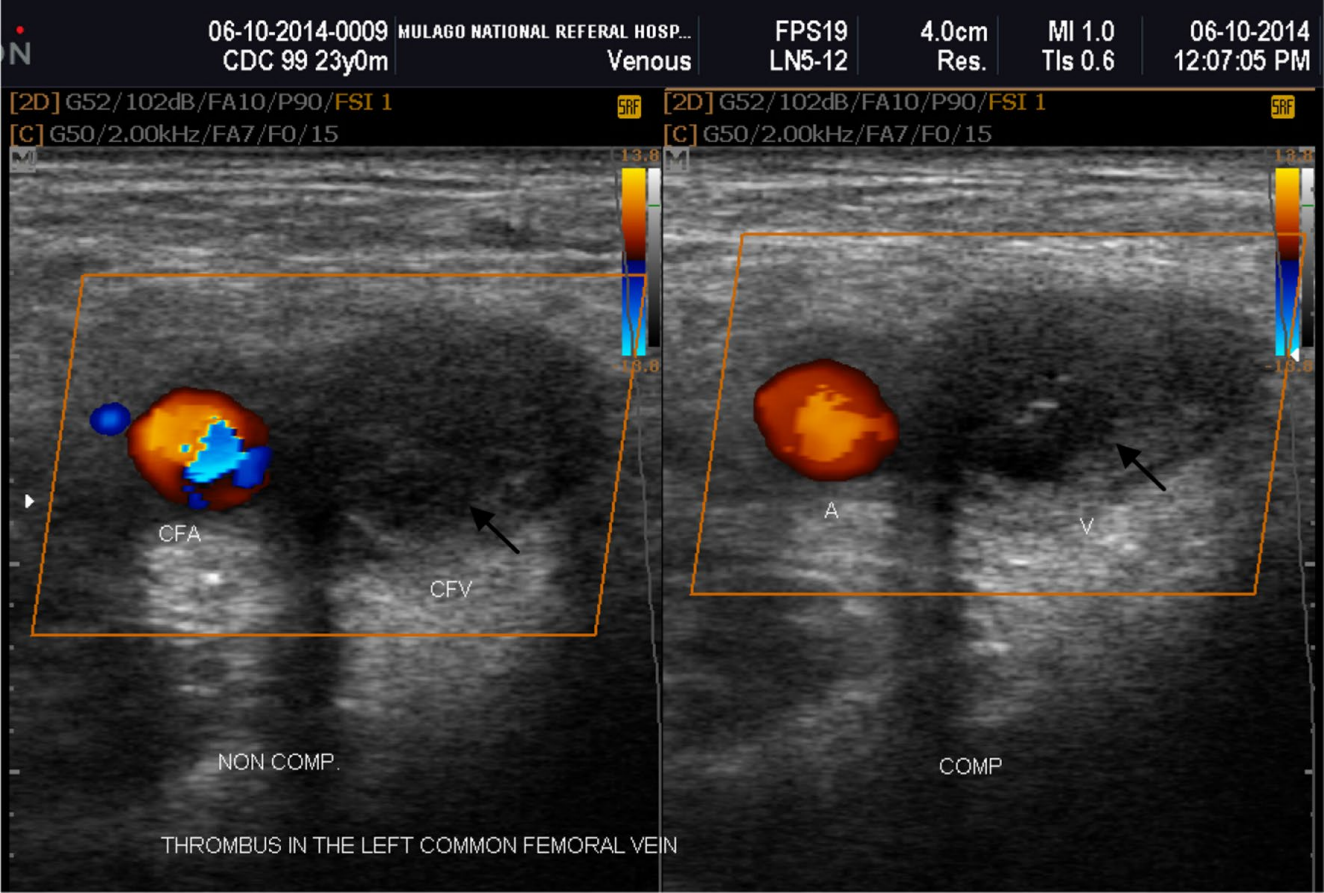
Acute DVT. Transverse sonogram of a 23 years old man on second line HAART who presented with acute DVT of CFV. Ultrasound scan shows lack of compressibility of the left CFV, presence of heterogeneous thrombus (black arrow) in the lumen and absence of spontaneous Doppler signal

The prevalence of lower limb DVT was 9.1% (35/384) (Table 2). Among 35 participants found with DVT, 26 (74.3%) were symptomatic and 9 (25.7%) participants asymptomatic for DVT. The prevalence of latent DVT among HIV patients on ART was 2.3%. Among 35 patients with DVT, 31.1% had acute DVT, 42.8% had chronic DVT, and the rest had latent DVT (Figs. 1, 2). Among the patients with DVT, 30 (80.7%) had proximal DVT and 5 had distal DVT. Bilateral deep venous thrombosis was noted in 3/35 (0.08%) participants.

**Table 2:** Prevalence of DVT in the study population

DVT	Type of DVT			
	n (%)	n (%)		
Present	35 (9.1)	Symptomatic for DVT	Acute	11 (31.4)
			Chronic	15 (42.9)
		Asymptomatic for DVT	Latent	9 (25.7)
Absent	349 (90.9)

Among participants who had DVT, 4 (11.4%) reported the use of hormonal contraception. Patients with hormonal contraception use had a high probability of DVT (p = 0.024).

On the Wells scoring out of 35 patients with DVT, 17 (48.6%) patients had a low probability of DVT, 8 (22.9%) patients had moderate probability of DVT and 10 patients had high probability of DVT. Study findings showed that among 35 patients with DVT, 23 (65.7%) patients had leg swelling (p < 0.0001).

In the bivariate analysis, study findings showed that prolonged immobility, platelets count, latest CD4 count, and ART line regimen had an association with deep venous thrombosis (see Table 3).

**Table 3:** Distribution of DVT by patient characteristics: bivariate analysis

Characteristic	Total population n (%) n = 384	DVT present n = 35 (9.1%)	DVT absent 349 (89.1%)	Chi square	p value
Gender				0.88	0.35
Male	126 (32.8%)	9 (25.7%)	117 (33.5%)		
Female	258 (67.2%)	26 (74.3%)	232 (66.5%)		
Age (years)				0.32	0.574
< 50	286 (74.5)	26 (74.3)	260 (74.5)	0.001	0.98
50 years and above	98 (25.5)	9 (25.7)	89 (25.5)		
Duration on ART (mean of years)	4.5 (2.5–8.5)	5.5 (2.5–8.5)	4.5 (2.5–8.5)		0.80
ART line status				6.17	*0.01*
First line	229 (59.6%)	14 (40.0%	215 (61.6%)		
Second line	155 (40.4%)	21 (60%)	134 (38.4%)		
Latest CD4 cell counts				6.73	*0.04*
Less than 200	23 (6.0%)	5 (14.3%)	18 (5.2%)		
200–500	194 (50.5%)	20 (57.1%)	174 (49.9%)		
Above 500	167 (43.5)	10 (28.6%)	157 (45.0%)		
Occupation				2.78	0.73
Business	148 (38.5%)	11 (31.4%)	137 (39.3%)		
Driver	18 (4.7%)	2 (5.7%)	16 (4.6%)		
Farmer	43 (11.2%)	5 (14.3%)	38 (10.9%)		
House wife	28 (7.3%)	3 (8.7%)	25 (7.2)		
Work in office	30 (7.8%)	1 (2.9%)	29 (8.3%)		
Others	117 (30.5%	13 (37.1%)	104 (29.8%)		
Prolonged immobility				10.21	*0.005*
Yes	11 (2.9%)	4 (11.4%)	7 (2.0%)		
No	373 (97.1%)	31 (88.6%)	342 (98.0%)		
Platelets count	235 (185–301)	206 (178–287)	236 (186–303)		0.10
BMI (median (IQR)	23.5 (21.5–26.9)	24.6 (21.9–28.1)	23.4 (21.5–26.8)		0.37

In the multivariate analysis (Table 4), the odds of occurrence of DVT among patients with prolonged immobility are 4.81 times as high as in those with no prolonged immobility. Being on second line ART regimen, is associated with higher odds of DVT occurrence compared with being on ART first line (OR = 2.38; 95% CI 1.14–4.97). Patients with a lower CD4 count (cd4 < 200 cells/µL) had 5.36 times the odds of DVT occurrence than those with higher CD4 count (cd4 > 500 cells/µL).

**Table 4:** Multivariate analysis: study findings

Characteristic	Crude odds ratio (95% CI)	p-value	Adjusted odds ratio (95% CI)	p-value
Gender				
Male	1.00		1.00	
Female	1.46 (0.66–3.21)	0.350	1.94 (0.84–4.47)	0.119
Age (years)				
< 50 years	1.00			
50 years and above	1.37 (0.45–4.15)	0.98		
Duration on ART (years)	1.02 (0.934–1.11)	0.679		
ART line status				
First line	1.00		1.00	
Second line	2.40 (1.18–4.89)	0.015	2.38 (1.14–4.97)	*0.020*
Latest CD4 count (cells/µl)				
Above 500	1.00		1.00	
200–500	1.80 (0.82–3.97)	0.143	1.82 (0.80–4.14)	0.153
Less than 200	4.36 (1.34–14.18)	0.014	5.31 (1.54–18.35)	*0.008*
Occupation				
Business	1.00			
Driver	1.56 (0.32–7.69)	0.586	–	–
Farmer	1.64 (0.54–5.00)	0.386	–	–
House wife	1.50 (0.39–5.74)	0.558	–	–
Work in office	0.43 (0.01–3.46)	0.430	–	–
Others	1.56 (0.68–3.61)	0.303	–	–
History of immobility				
No	1.00		1.00	
Yes	6.30 (1.75–22.72)	0.005	4.81 (1.25–18.62)	*0.023*
Platelets count	0.99 (0.96–1.00)	0.103	0.99 (0.97–1.02)	0.110
Body mass index	1.02 (0.94–1.09)	0.687	–	–

## Discussion

We found the prevalence of DVT in ambulatory HIV positive patients on ART to be 9.1% at the National Referral Hospital, Kampala, Uganda. This prevalence is higher than previously reported in several studies among HIV-infected patients in Western and African countries ranging from 0.19 to 8% per year [6, 9–11] The prevalence of DVT in HIV-infected patients is within limits from previous studies although a little higher than in the general population in Uganda [12] (1/1000 person-years of observation). This is especially striking as these patients were attending for routine follow up for their HIV and were not admitted as in-patients or attending hospital because they were unwell. To our knowledge, this is the first study to determine the prevalence of DVT among HIV sero-positive out patients on ART in Eastern Africa.

In this study, 15 of 35 patients found with DVT had hyperechoic thrombus (42%). The other sonographic characteristic of DVT noted in this study were the same characteristics reported in other studies [13, 14].

Majority of participants found with DVT had clinical symptoms of this pathology (Table 2) and only few patients had latent DVT (2.3%). Almost half of HIV patients with DVT (48.6%) had a lower Wells score for DVT (Table 3). This suggests that DVT in this population is difficult to predict clinically therefore Well’s score need to be altered to account for HIV infection although other studies have shown that patients with high score are likely to develop DVT [5]. This study demonstrates that infected HIV patients on ART may be asymptomatic for DVT according Wells score findings however they may develop an asymptomatic DVT. Based on the results of Wells score in this study, a compression ultrasound screening in HIV positive patients on ART may be helpful for early diagnosis.

There was a significant association between PIs containing second line ART and deep venous thrombosis in patients (p = 0.02) which is in keeping with several other studies. HIV-infected patients on second line ART regimen (PIs) have higher risk for blood clot formation than those on first line treatment [5, 8, 15, 16]. Studies supporting this association reported high rate of DVT among HIV-infected patients after the introduction of protease inhibitors in ART regimen [4].

This study shows significant association between hormonal contraception use and DVT (p = 0.024) as per well known evidence for increased risk of VTE associated with combined hormonal contraceptive use generally [22].

Eleven (2.9%) of 384 participants had history of immobility in the last 3 months. Among 35 participants with DVT, 4 (11.4%) had history of immobility (p = 0.005). This is also in line with other studies suggesting that chronic immobility (> 30 days) is a high risk for deep venous thrombosis [17–19]. These study findings were similar to the case–control study in 2008 conducted by Leibson and colleagues in 2008 [20, 21]. HIV patients are susceptible to develop opportunistic infections responsible of hospitalization, and this will put them at increased risk for DVT [15, 21–23].

Our study showed that among 23 participants (6%) with low CD4 cell counts (< 200 cells/mm^3^), 5 participants (14.3%) had DVT. Therefore, correlation between low CD4 and DVT was significant even after correcting for immobility which may be associated with higher rates of opportunistic infections in people with low CD4 counts. Several studies reported the same findings [5–24]. The association between low CD4 count and DVT may be related to progressive immunosuppression and increasing hypercoagulable state [25]. Having a low CD4 count can be associated with having an uncontrolled high HIV viral load. HIV with a high viral load is associated with hypercoagulable state. Unfortunately, viral load was not done in all patients recruited in this study therefore it was not possible to analyse any association between DVT and viral load.

Whilst newer lower molecular weight anti-coagulation is expensive for low resource settings, traditional anti-coagulation with warfarin is cheap, although logistically difficult. Research into the cost–benefit of use of newer anti-coagulation or point of care monitoring of warfarin in patients at highest risk of DVT in our setting should be considered. The blood tests such as D-dimer, tissue factor (TF) and analysis of other coagulation biomarkers should also be considered in relation to blood clot formation in this population with hypercoagulable state.

## Conclusion and recommendations

The prevalence of DVT among ambulatory HIV positive patients on ART is 9.1%. Majority of participants with DVT were symptomatic. Prolonged immobility, low CD4 count (< 200 cells/µl) and ART second line (PIs) were associated with risk of DVT. It is advisable to perform lower limb Doppler ultrasound scan examination on infected HIV patients on ART who are symptomatic for DVT. However, half of patients with a DVT had a low Wells score suggesting that they did not have typical clinical features of DVT. Therefore, clinicians should consider prophylactic anti-coagulation or routine Doppler ultrasound scan monitoring in HIV patients with low CD4 counts and PIs who are immobile knowing that they may develop an asymptomatic DVT. Patients using oral contraception on PIs and with low CD4 count should be counselled on signs and symptoms of DVT that they should report to their doctor and should be monitored closely for DVT.

## Abbreviations

ART: antiretroviral therapy
DVT: deep venous thrombosis
HAART: highly active antiretroviral therapy
HIV: human immunodeficiency virus
IDI: infectious disease institute
PE: pulmonary embolism
PIs: protease inhibitors
VTE: venous thromboembolism
